# Performance of serological tests available in Brazil for the diagnosis of human visceral leishmaniasis

**DOI:** 10.1371/journal.pntd.0007484

**Published:** 2019-07-18

**Authors:** Mariana Lourenço Freire, Tália Machado de Assis, Edward Oliveira, Daniel Moreira de Avelar, Isadora C. Siqueira, Aldina Barral, Ana Rabello, Gláucia Cota

**Affiliations:** 1 Pesquisa Clínica e Políticas Públicas em Doenças Infecciosas e Parasitárias, Instituto René Rachou, Fundação Oswaldo Cruz, Belo Horizonte, Minas Gerais, Brasil; 2 Centro Federal de Educação Tecnológica de Minas Gerais, Contagem, Minas Gerais, Brasil; 3 Laboratório de Patologia Experimental, Instituto Gonçalo Muniz, Fundação Oswaldo Cruz, Salvador, Bahia, Brasil; 4 Laboratório de Enfermidades Infecciosas Transmitidas por Vetores, Instituto Gonçalo Muniz, Fundação Oswaldo Cruz, Salvador, Bahia, Brasil; US Food and Drug Administration, UNITED STATES

## Abstract

**Background:**

Visceral leishmaniasis (VL) is the most severe form of leishmaniasis and is potentially fatal if not diagnosed and treated. Accurate and timely diagnosis is considered one of the pillars needed for the reduction in disease-related lethality. Brazil is currently one of the three eco-epidemiological hotspots for this disease. Several serological tests are commercially available in this country for VL diagnosis, although information on the performance of these tests is fragmented and insufficient. The aim of this study was to directly compare the performance of six commercial kits: three enzyme-linked immunosorbent assays (ELISAs), two immunofluorescence antibody tests (IFATs), one immunochromatographic test (ICT), besides one ICT, currently not commercially available in Brazil and one *in-house* direct agglutination test (DAT-LPC), not yet marketed.

**Methodology/Principal findings:**

A panel of 236 stored samples from patients with clinically suspected VL, including 77 HIV-infected patients, was tested. IT-LEISH and DAT-LPC showed the highest accuracy rate among the non-HIV-infected patients, 96.2% [CI95%: 92.8–99.7%] and 95.6% [CI95%: 91.9–99.3%], respectively. For the ELISA tests evaluated, the maximum accuracy was 91.2%, and in the inter HIV-status group analysis, no significant differences were observed. For both IFATs evaluated, the maximum accuracy was 84.3%, and a lower accuracy rate was observed among the HIV-infected patients (p = 0.039) than among the non-HIV-infected patients. The DAT-LPC was the most accurate test in the HIV-infected patients (p≤0.115). In general, no significant difference in accuracy was observed among the VL-suspected patients stratified by age.

**Conclusions/Significance:**

In summary, the differences in the performance of the tests available for VL in Brazil confirm the need for local studies before defining the diagnostic strategy.

## Author summary

Visceral leishmaniasis (VL) is a tropical disease distributed worldwide. In the Americas, Brazil reports about 96% of VL cases, which has been highlighted by the increase in lethality in last years. Accurate VL diagnosis is of utmost importance. Despite this, the performance of some commercial tests currently available in Brazil is unknown, especially for HIV-infected patients. Accordingly, in this study we present a comparative performance analysis of six commercial kits available in Brazil for the diagnosis of VL in non-HIV and HIV-infected patients, besides one immunochromatographic test (ICT) and one *in-house* direct agglutination test (DAT-LPC) currently not commercially available in Brazil. ICTs and DAT-LPC showed better performance among non-HIV infected patients. Despite the known limitation of serological tests for the diagnosis of patients with HIV, the direct agglutination test was more accurate in this specific group of patients. Our results demonstrate significant differences in the performance of different serological tests and confirm that the use of serology should be qualified with previous information on the performance of the tests.

## Introduction

Human visceral leishmaniasis (VL) is a neglected tropical disease (NTD) endemic to more than 65 countries with an average of 25,000 new cases reported per year from 2013–2017. Over 90% of global VL cases were reported from seven countries: Brazil, Ethiopia, India, Kenya, Somalia, South Sudan and Sudan. If left untreated, VL is fatal in more than 95% of the cases within two years after the onset of the disease [1]. Leishmaniasis is linked to environmental changes such as deforestation, building of dams, irrigation schemes and urbanization. For these reasons, despite the advances in diagnosis and even with taking the successfully implemented control programmes into account, in recent years, the disease has expanded mainly on the Indian continent [2,3].

Approximately 96% of the VL cases in South America are reported in Brazil, with an average of 3,749 cases reported annually with a lethality of 6.9% [4,5]. Since the clinical features of VL mimic several other common diseases and the treatment is associated with significant toxicity, an accurate diagnosis is crucial. The gold standard for VL diagnosis remains the demonstration of *Leishmania* parasites, in bone marrow aspirate or in other biologic specimens, as spleen or liver. This strategy requires expertise of health professionals to perform both the biological sample collection and execution of parasitological exam [6]; in addition to a time from hours to days, depending on the conditions of the service, until the release of the result.

On the other hand, a wide range of serological tests are available and are considered the main tools for the diagnosis of VL. Serology exhibits variable performance in diagnosis of VL depending upon antigens, and immune status of the human host. In this sense, the human immunodeficiency virus (HIV) associated with *Leishmania* infection represents a further challenge due to the reduction in the antibody levels in this subgroup of patients [7]. In turn, immunochromatographic tests using recombinant K39 antigen (rK39-ICT) represent a breakthrough in VL diagnostics in recent years because of the high performance and low cost of the test, coupled with the fast and easy execution profile [8].

The Brazilian Visceral Leishmaniasis Surveillance and Control Programme have been providing the immunofluorescence antibody test (IFAT) for the diagnosis of VL in recent decades. In 2009, the first rK39-ICT was incorporated, and since then, the brand has already been replaced twice. In parallel, several other commercial tests based on enzyme-linked immunosorbent assays (ELISAs) and IFAT are registered in the national agency—ANVISA (Agência Nacional de Vigilância Sanitária)—that regulates the commercialization of diagnostic kits, and are widely used, mainly in the private health sector [9]. No commercial test based on direct agglutination test (DAT) is available in Brazil for the diagnosis of VL, but this test has been improved and has been used with good results in research [10,11]. Despite this diversity of serological tests for VL diagnosis, no study so far has comparatively evaluated the performance of these tests under the same conditions and in the same population. The goal of this study is to present a comparative analysis of the performance of the serological tests available for VL diagnosis in Brazil.

## Materials and methods

This diagnostic accuracy study was conducted with a stored panel of serum samples collected from patients with suspected VL. The study complies with the updated Standards for Reporting of Diagnostic Accuracy (STARD) statement [12]. A flow diagram that describes the design of the study according to the STARD statement is presented in S1 Fig and the STARD checklist in S1 Checklist.

### Study site

The study was conducted at the Laboratory of Clinical Research and Public Policy in Infectious and Parasitic Diseases at the Instituto René Rachou of the Oswaldo Cruz Foundation (IRR/Fiocruz)–a national reference centre for leishmaniasis in Belo Horizonte in the state of Minas Gerais, Brazil. The tested samples came from patients with suspected VL residing in three VL endemic Brazilian states: Minas Gerais (Southeastern region), Piauí and Bahia (Northeastern region).

### Characteristics of sera and study design

A range of 236 serum samples stored at -70°C from patients with clinical manifestations compatible with VL (including HIV co-infected patients) recruited in previous clinical studies was used to assess the tests’ performance. The sample size estimation was calculated using Stata software version 9.2 (STATA Corporation, College Station, Texas, USA) following the rationale for the two main objectives of the study, in both cases using a power of 80% and an alpha error of 5%: I. to assess the performance of tests among patients without immunodeficiency, according to the performance proposed as the minimum required for a VL serological test: around 95% for sensitivity [13], a minimum one-sample size of 150 samples for proportion comparison was estimated; II. to compare the performance of the tests between HIV infected and uninfected patients, for the identification of a difference equal to or greater than 10% between the groups, an estimated size of 72 samples (in each group) was calculated for two-sample comparison of proportions.

All sample included in this study were derived from patients presenting clinical suspicion of VL. According to the Brazilian definition [14], a suspected VL case has fever and at least one clinical sign as splenomegaly, hepatomegaly, leukopenia, anaemia or thrombocytopenia. The samples from patients with a previous history of VL were excluded as well as the serum samples with aliquots with insufficient volume to perform all the tests.

The samples were divided into VL cases and non-cases. The criteria for the VL case definition was based on parasitological confirmation of *Leishmania* infection in the bone marrow aspirate (118 samples) and for the non-VL cases, a negative parasitological examination with the confirmation of another disease, such as malaria, schistosomiasis, mycobacteria infection and leukemia (118 samples). In total, 77 samples from HIV-infected patients were included in this study, being 38 in the VL cases group and 39 in the non VL cases group. All the samples were anonymized, and the diagnostic test operators were blinded to the nature of the serum sample.

### Serological diagnostic tests

At the first stage of this study, in January 2017, a search for the tests registered for VL diagnosis was performed via the electronic database of the Brazilian agency for registration of health products, ANVISA [9]. Then, the commercial availability of each product in Brazil was checked with the manufacturer or its legal distributor. Six kits (three ELISA kits, two IFAT kits and one rK39-ICT) were identified. Even without registration in force in Brazil, we chose to include Kalazar Detect, the first rapid test used in Brazil between 2009 and 2014. In the same way, the prototype of a direct agglutination test (DAT) produced in our laboratory (DAT-LPC)–a non-commercial kit–was also included among the tests to be evaluated, a decision based on the promising results observed in several validation studies previously performed [10,15]. In the end, eight test kits were selected for testing in this study, and their characteristics are listed in Table 1. The test’s manufacturers had no role in study design, analysis, decision to publish, or preparation of the manuscript. All tests were performed in strict accordance with the manufacturer’s instructions. The prototype DAT-LPC kit was performed as described by Oliveira et al. (2017) [10].

**Table 1:** Description of the diagnostic kits for human visceral leishmaniasis included in the study.

Diagnostic kits	Manufacturer	Method	Country	Commercial availability	Antigen
***Leishmania* ELISA IgG+IgM**	Vircell S. L.	ELISA	Spain	Yes	*L*. *infantum*
**Ridascreen *Leishmania* Ab**	R-Biopharm AG	ELISA	Germany	Yes	*L*. *infantum*
**NovaLisa*Leishmania infantum* IgG**	Novatec Immundiagnostica GMBH	ELISA	Germany	Yes	*L*. *infantum*
**IFI Leishmaniose Humana**	Fundação Oswaldo Cruz	IFAT	Brazil	Yes	Promastigotes of *Leishmania* spp.
***Leishmania* IFA IgG**	Vircell S. L.	IFAT	Spain	Yes	Promastigotes of *L*. *infantum*
**IT LEISH**	BIO-RAD Laboratories, Inc.	rK39-ICT	France	Yes	rK39
**Kalazar Detect**	Inbios International, Inc.	rK39-ICT	United States	No	rK39
**DAT-LPC**	IRR/Fiocruz	DAT	Brazil	No	Promastigotes of *L*. *infantum*

### Ethics statement

The study was carried out in conformity with the Helsinki Declaration and the Brazilian rules (RDC 466/2012). Ethical approval was obtained from the Research Ethics Committee of IRR/FIOCRUZ (CAEE 44549915.2.0000.5091 –Approval number 1.808.889). During the original clinical studies, written informed consent was obtained from all the participants/parents or guardians before collecting samples. Confidentiality was assured by assigning a study code to each sample, and no confidential information was shared.

### Statistical analysis

Data analysis was performed using MedCalc for Windows, version 15.0 (MedCalc Software, Ostend, Belgium) and IBM SPSS Statistics (Chicago, IL, USA) software. Sensitivity, specificity and accuracy were calculated using two-by-two contingency table with exact binomial 95% confidence interval (95% CI), and explored in different age and HIV-status groups. Considering sensitivity as the probability of being a test positive when disease is present, the sensitivity rate was calculated as the number of patients with VL who tested positive divided by the total number of patients with VL. Considering specificity as the probability of being a test negative when disease is absent, the specificity rate was calculated as the number of non-VL patients who tested negative divided by the total number of non-VL patients. The accuracy rate is proportion of patients presenting a correct test result and was calculated as the number of patients with VL who tested positive plus the number of non-VL patients who tested negative divided by the total number of patients tested. These parameters were compared using the χ^2^ test at a significance level of 0.05. We used the Cohen kappa index for agreement testing between the test results. The values of the Cohen κ coefficients were interpreted according to Landis and Koch: 1.00–0.81: excellent, 0.80–0.61: good, 0.60–0.41: moderate, 0.40–0.21: weak and 0.20–0.00: negligible agreement.

## Results

### Participants’ characteristics

Sixty-six percent of the 236 suspected VL cases were male, with an average age of 25±19.7 years (range: 1 month to 76 years). Among 159 non-HIV-infected patients, the mean age was 18±19.14 years, and 40 patients (25.2%) were under 3 years. In the HIV-infected group, the mean age was 40 ± 9.8 years (ranging from 20 to 65 years), and 74% of the patients were male. No statistically significant difference was observed between the accuracy of the tests according to the gender of the patients.

### Performance of different diagnostic kits according to HIV status

The sensitivity, specificity and accuracy of the VL tests according to the patient’s HIV status are shown in Table 2. The tests evaluated exhibited lower sensitivity rates among the HIV-infected patients than among the non-HIV-infected patients (p≤0.05), except for the *Leishmania* ELISA IgG+IgM test (p = 0.104) and the DAT-LPC test (p = 0.412). Furthermore, when the accuracy rates were compared, significant differences were observed for the NovaLisa *Leishmania infantum* IgG test (p = 0.019), *Leishmania* IFA IgG test (p = 0.035), IT-LEISH test (p = 0.001) and Kalazar Detect test (p≤0.0001), which also exhibited significantly lower accuracy among the HIV-infected patients than among the non-HIV-infected patients. The agreement among all tests evaluated for HIV-infected and HIV-uninfected patients was calculated by Cohen kappa index (S1 Table).

**Table 2:** Performance of the human visceral leishmaniasis diagnostic kits stratified according to HIV status.

**Diagnostic kits**	**NON-HIV-INFECTED PATIENTS**		
	**Sensitivity (%) [CI 95%]** **(n = 80)**	**Specificity (%) [CI 95%]** **(n = 79)**	**Accuracy (%) [CI 95%]**
***Leishmania* ELISA IgG+IgM**	77.5 [67.2–85.3] (62/80)	93.7 [86.0–97.3] (74/79)	85.5 [79.2–90.2]
**Ridascreen *Leishmania* Ab**	93.8 [86.2–97.3] (75/80)	77.2 [66.8–85.1] (61/79)	85.5 [79.2–90.2]
**NovaLisa *Leishmania infantum* IgG**	86.3 [77.0–92.2] (69/80)	96.2 [89.4–98.7] (76/79)	91.2 [85.8–94.7]
**IFI Leishmaniose Humana**	86.3 [77.0–92.2] (69/80)	82.3 [72.4–89.1] (65/79)	84.3 [77.8–89.1]
***Leishmania* IFA IgG**	78.8 [68.6–86.3] (63/80)	96.2 [89.4–98.7] (76/79)	87.4 [72.7–88.3]
**IT LEISH**	96.3 [89.6–98.7] (77/80)	96.2 [89.4–98.7] (76/79)	96.2 [92.0–98.3]
**Kalazar Detect**	92.5 [84.6–96.5] (74/80)	94.9 [87.7–98.0] (75/79)	93.7 [88.8–96.6]
**DAT-LPC**	93.8 [86.2–97.3] (75/80)	97.5 [91.2–99.3] (77/79)	95.6 [91.2–97.9]
**Diagnostic kits**	**HIV INFECTED PATIENTS**		
	**Sensitivity (%) [CI 95%]** **(n = 38)**	**Specificity (%) [CI 95%]** **(n = 39)**	**Accuracy (%) [CI 95%]**
***Leishmania* ELISA IgG+IgM**	63.2 [47.3–76.6] (24/38)	97.4 [86.8–99.6] (38/39)	80.5 [70.3–87.8]
**Ridascreen *Leishmania* Ab**	78.9 [63.7–88.9] (30/38)	89.7 [76.4–95.9] (35/39)	84.4 [74.7–90.9]
**NovaLisa *Leishmania infantum* IgG**	65.8 [49.9–78.8] (25/38)	94.9 [83.1–98.6] (37/39)	80.5 [70.3–87.8]
**IFI Leishmaniose Humana**	60.5 [44.7–74.4] (23/38)	89.7 [76.4–95.9] (35/39)	75.3 [64.7–83.6]
***Leishmania* IFA IgG**	60.5 [44.7–74.4] (23/38)	92.3 [79.7–97.4] (36/39)	76.6 [66.1–84.7]
**IT LEISH**	63.2 [47.3–76.6] (24/38)	97.4 [86.8–99.6] (38/39)	80.5 [70.3–87.8] *
**Kalazar Detect**	47.4 [32.5–62.7] (18/38)	97.4 [86.8–99.6] (38/39)	72.7 [61.9–81.4] #
**DAT-LPC**	89.5 [75.9–95.8] (34/38)	89.7 [76.4–95.9] (35/39)	89.6 [80.8–94.6]

CI95%: 95% confidence interval

#### Enzyme-linked immunosorbent assays

Among the non-HIV-infected patients, the sensitivity for ELISA tests varied from 77.5% [CI95%: 67.2–85.3%] to 93.8% [CI95%: 86.2–97.3%]. The highest value of sensitivity was obtained with the Ridascreen *Leishmania* Ab test, the only ELISA test that presented a positive assertiveness rate over 90%. However, the specificity rate of this test– 77.2% [CI95%: 66.8–85.1]–was the lowest among the other ELISAs (p<0.003); therefore, the accuracies of these tests were not significantly different. Among the HIV-infected patients, no significant differences were observed for sensitivity (p≥0.134), specificity (p≥0.170) or accuracy (p≥0.655) rates. Considering the inter-HIV status group analysis, no significant differences were observed in the accuracy rates among the three ELISA tests evaluated (p>0,114). A good agreement was observed among the ELISA tests according to the Cohen kappa index (K≥0.65 [CI95%:0.53–0.76]).

#### Immunofluorescence antibody tests

Considering the intra HIV-status groups, the two IFAT tests showed similar sensitivity (p≥0.213) and accuracy (p≥0.428) rates. For non-HIV-infected patients, *Leishmania* IFA IgG exhibited higher specificity (p = 0.005) than *Leishmania* IFA IgG. A moderate agreement between the two IFATs was observed–K = 0.54 [CI95%: 0.41–0.67]–among the non-HIV-infected patients and a good agreement–K = 0.63 [CI95%: 0.44–0.81]–was observed among the HIV-infected patients.

#### Immunochromatographic tests

For both rapid tests, a significant decrease in the sensitivity and accuracy rates was observed in the HIV-infected group compared to those of the non-HIV-infected patients (p≤0.0001). For the non-HIV-infected patients, the estimated sensitivities of the IT-LEISH and Kalazar Detect were 96.3% [CI95%: 89.6–98.7%] and 92.5% [CI95%: 84.6–96.5%], respectively. In the HIV-infected patients, the sensitivity dropped to 63.2% [CI95%: 47.3–76.6%] and to 47.4% [CI95%: 32.5–62.7], respectively. In the intra HIV-status groups, no significant differences were observed for the sensitivity, specificity and accuracy rates between the two rK39-ICTs (p>0,169). An excellent and good agreement between the rK39-ICT among the non-HIV-infected patients was observed (K = 0.90 [CI95%: 0.83–0.97]) and among the HIV-infected patients (K = 0.68 [CI95%: 0.51–0.86]), respectively. Four false-positive results were found for Kalazar Detect (patients with sepsis, infective endocarditis, lymphoma, and hepatic insufficiency); two of these patients also tested positive by the IT-LEISH. Among the HIV-infected group, one false-positive result occurred in a patient with mycobacteria infection.

#### Direct agglutination test

For non-HIV-infected patients, the estimated sensitivity was 93.8% [CI95%: 86.2–97.3], and in HIV-infected patients, the sensitivity was 89.5% [CI95%: 75.9–95.8], with a non-significant statistical difference (p = 0.412). Among the 118 non-VL samples, DAT-LPC presented six false-positive results, half of them from patients with other infectious diseases. The higher agreement index with the DAT-LPC, among the non-HIV-infected patients, was observed for both the IT-LEISH and NovaLisa *Leishmania infantum* IgG tests (K = 0.86 [CI95%: 0.78–0.94]).

### Performance of different diagnostic kits for non-HIV-infected patients according to the age stratification

Table 3 shows the VL tests’ performance for the HIV-uninfected group stratified according to age. The sensitivity, specificity and accuracy rates of the testes were no statistically different in the comparison between patients grouped by age, except for Kalazar Detect, which exhibited significantly lower sensitivity among the children under 3 years old compared to patients upper 3 years old: 86.2% [CI95%:69.4–94.5] *versus* 96.1% [CI95%:86.78–98.9], p = 0.046.

**Table 3:** Performance of the human visceral leishmaniasis diagnostic kits for non-HIV-infected patients according to age stratification.

**Diagnostic kits**	**0 to 3 years**		
	**Sensitivity (%) [CI 95%]** **(n = 29)**	**Specificity (%) [CI 95%]** **(n = 11)**	**Accuracy (%) [CI 95%]**
***Leishmania* ELISA IgG+IgM**	75.9 [57.9–87.8] (22/29)	90.9 [62.3–98.4] (10/11)	80.0 [65.2–89.5]
**Ridascreen *Leishmania* Ab**	93.1 [78.0–98.1] (27/29)	81.8 [52.3–94.9] (09/11)	90.0 [77.0–96.0]
**NovaLisa *Leishmania infantum* IgG**	89.7 [73.6–96.4] (26/29)	100.0 [74.1–100.0] (11/11)	92.5 [80.1–97.4]
**IFI Leishmaniose Humana**	89.7 [73.6–96.4] (26/29)	72.7 [43.4–90.3] (08/11)	85.0 [70.9–92.9]
***Leishmania* IFA IgG**	75.9 [57.9–87.8] (22/29)	100.0 [74.1–100.0] (11/11)	82.5 [68.1–91.3]
**IT LEISH**	93.1 [78.0–98.1] (27/29)	100.0 [74.1–100.0] (11/11)	95.0 [83.5–98.6]
**Kalazar Detect**	86.2 [69.4–94.5] (25/29)	90.9 [62.3–98.4] (10/11)	87.5 [73.9–94.5]
**DAT–LPC**	93.1 [78.0–98.1] (27/29)	100.0 [74.1–100.0] (11/11)	95.0 [83.5–98.6]
**Diagnostic kits**	**Over 3 years**		
	**Sensitivity (%) [CI 95%]** **(n = 51)**	**Specificity (%) [CI 95%]** **(n = 68)**	**Accuracy (%) [CI 95%]**
***Leishmania* ELISA IgG+IgM**	78.4 [65.37–87.5] (40/51)	94.1 [85.8–97.7] (64/68)	87.4 [80.2–92.2]
**Ridascreen *Leishmania* Ab**	94.1 [84.08–98.0] (48/51)	76.5 [65.1–85.0] (52/68)	84.0 [76.4–89.5]
**NovaLisa *Leishmania infantum* IgG**	84.3 [71.99–91.8] (43/51)	95.6 [87.8–98.5] (65/68)	90.8 [84.2–94.8]
**IFI Leishmaniose Humana**	84.3 [71.99–91.8] (43/51)	83.8 [73.3–90.7] (57/68)	84.0 [76.4–89.5]
***Leishmania* IFA IgG**	80.4 [67.54–89.0] (41/51)	95.6 [87.8–98.5] (65/68)	89.1 [82.2–93.5]
**IT LEISH**	98.0 [89.7–99.7] (50/51)	95.6 [87.8–98.5] (65/68)	96.6 [91.7–98.7]
**Kalazar Detect**	96.1 [86.78–98.9] (49/51)	95.6 [87.8–98.5] (65/68)	95.8 [90.5–98.2]
**DAT–LPC**	94.1 [84.08–98.0] (48/51)	97.1 [89.9–99.2] (66/68)	95.8 [90.5–98.2]

CI95%: 95% confidence interval

## Discussion

The main contribution of this study is the confirmation of significant differences in the performances of different serological tests available for VL diagnosis in Brazil. This observation confirms that the recommendation of the use of serology as one of the main diagnostic strategies of the national programme to achieve lethality reduction needs to be qualified with specific information about the test performance. There is no consensus about the minimum sensitivity and specificity rates required for a VL diagnostic test. According to Boelaert et al. (2007) [13], for a VL-screening test, the minimum sensitivity and specificity required would be 95% and 98%, respectively. Considering these parameters, none of the diagnostic tests evaluated here are satisfactory. In this sense, questions emerge from these results surrounding the adequacy (or inadequacy) of the current tests as efficient tools for tracking the disease. Another contribution of this work is to demonstrate variations in test performance when applied to different populations through a direct test comparison using a well-defined panel of samples controlling for the heterogeneity of this population under the same conditions. Our results confirm that the differences in test performance are related to the test’s methodology and to the HIV infection status and age of patients. From these, the HIV co-infection was the factor that more impacted in the performance of the serological tests. The higher frequency of false-negative results in the VL/HIV co-infected patients may be explained by the functional impairment of cell-mediated immunity due to viral infection that result in the absence or lower response to *Leishmania* spp. infection [16].

In this study, the tests that presented the best performance for the diagnosis of VL among the non-HIV-infected patients were IT-LEISH, DAT-LPC and Kalazar Detect, each with a sensitivity and specificity above 90%. These results are in agreement with those found in a meta-analysis involving populations mainly infected by *Leishmania donovani*, which estimated the sensitivity of the rK39-ICT and DAT to be 93.9% and 94.8%, respectively [17].

Concerning rK39-ICT, it is widely known that there are differences in the performance of these tests in different endemic regions as well as among tests produced by different manufacturers in the same endemic region [18–21]. Here, no significant differences were observed between the accuracy of the IT-LEISH and Kalazar Detect tests, independent of the HIV infection status. However, an unsatisfactory rK39-ICT performance was confirmed among the HIV-infected individuals (63.2% and 47.4% sensitivity for IT-LEISH and Kalazar Detect, respectively), similar to the results described in other studies [15,21–24]. This finding overrides the potential advantages of a rapid, inexpensive and easy-to-perform test, making this diagnostic strategy insufficient for HIV co-infected or unknown HIV status patients. On the other hand, the high performance of the DAT in this patient group has been systematically reported, with sensitivity varying from 87.8 to 91.7% and specificity varying from 82.3 to 83.3% [15,22–24], similar to that observed here–a DAT-LPC sensitivity of 89.5% and specificity of 89.7%.

Although ELISAs and IFATs are important diagnostic techniques for infectious diseases in general, we verified an unsatisfactory accuracy of these tests for the diagnosis of VL. For non-HIV-infected patients, the IFATs and ELISAs showed the lowest performance among all the tests evaluated, especially in sensitivity, as demonstrated by Mniouil et al. (2018) and Mikaeili et al. (2007) [25,26]. In contrast, the Ridascreen *Leishmania* Ab test exhibited a higher sensitivity (93.8%) compared to that of the other ELISA tests evaluated here, which was also observed in other studies [25,27,28]. However, the low specificity (77.2%) of the Ridascreen *Leishmania* Ab test results in a low final accuracy. For the IFATs, both tests evaluated exhibited insufficient accuracy, similar to that reported by Pedras et al. (2008) and Bangert (2018) [24,27]. It is important to note that the IFATs evaluated here use distinct species of *Leishmania*-promastigote forms as antigens: *Leishmania major* in IFI-Leishmaniose Humana and *Leishmania infantum* in Leishmania IFA IgG, which apparently did not impact their low performance. The insufficient IFATs’ performance, particularly that of IFI-Leishmaniose Humana, needs to be highlighted in Brazil, since this test is still available to the public health service and is widely used[4].

The variation in the test performance according to the patient's age was explored by testing several age cut-off points. The accuracy of the Kalazar Detect test was significant lower among children under 3 years old comparison to patients over three years old. Few studies have evaluated the performance of rapid tests in very young paediatric populations. In the study conducted by Cruz et al., the performance of the rapid test did not differ between individuals over 10 years in age in relation to children of up to 10 years in age [29]. Overall, in this study, the observed sensitivity was higher than we have shown (75.9 versus 93.1%), which may be explained by differences in the case definition criteria and by the higher age of the children evaluated.

The use of accurate diagnostic tests is especially important for VL, a disease in which the misdiagnosis could be extremely dangerous in both scenarios: a false-positive result would lead to an unnecessary toxic treatment and a false negative test result would leave untreated patients with a lethal disease. The performances of serological diagnostic tests are expected to vary with the methodology of the test, the type of antigen, infection length and characteristics of the individual, such as immune status and age. In the case of VL, a disease with a global distribution, several of these determinants act simultaneously to influence the performance of the tests. In summary, these results have direct implication in public health care policies in Brazil. In addition to confirming the high performance of rapid immunochromatographic tests in general, the results show important exceptions, people living with HIV and children younger than 3 years old, specific groups in which a rapid test cannot be used to rule out the VL diagnosis safely. For these groups, a specific algorithm is required, and DAT-LPC emerges as the best performing serological test, which adds to its advantage in terms of national autonomy in production. In addition, these results suggest the presence of significant differences in the performance of tests from different manufacturers using the same methodology, which reinforces the need for local validations of the different tests before their use in large scale. Our findings highlight the need for more stringent criteria for the registration of diagnostic products in Brazil, including the requirement to carry out validation studies before marketing. In a future, broader analysis, in addition to performance, other aspects of these tests should be considered before a diagnostic strategy is defined, such as cost-effectiveness, national production/autonomy and accessibility. In this context, this study represents the first step of a wider evaluation required.

## Supporting information

S1 Checklist STARD checklist for reporting of studies of diagnostic accuracy. The STARD checklist describes the design of the current study in order to improve reporting accuracy and completeness.
(DOCX)

S1 Fig STARD flow diagram for reporting of studies of diagnostic accuracy. P: positive; N: negative; ID: indeterminate; TC: target condition.
(TIF)

S1 Table Concordance analysis of commercially available diagnostic kits for human visceral leishmaniasis in Brazil. The concordance analysis was calculated through Cohen kappa index to demonstrate the agreement between the test’s results.
(DOCX)
