# Supplementary figures and images for: Performance of serological tests available in Brazil for the diagnosis of human visceral leishmaniasis
Source: PLoS Negl Trop Dis. 2019 Jul 18;13(7):e0007484. doi: 10.1371/journal.pntd.0007484 (PMC6638734; doi:10.1371/journal.pntd.0007484)

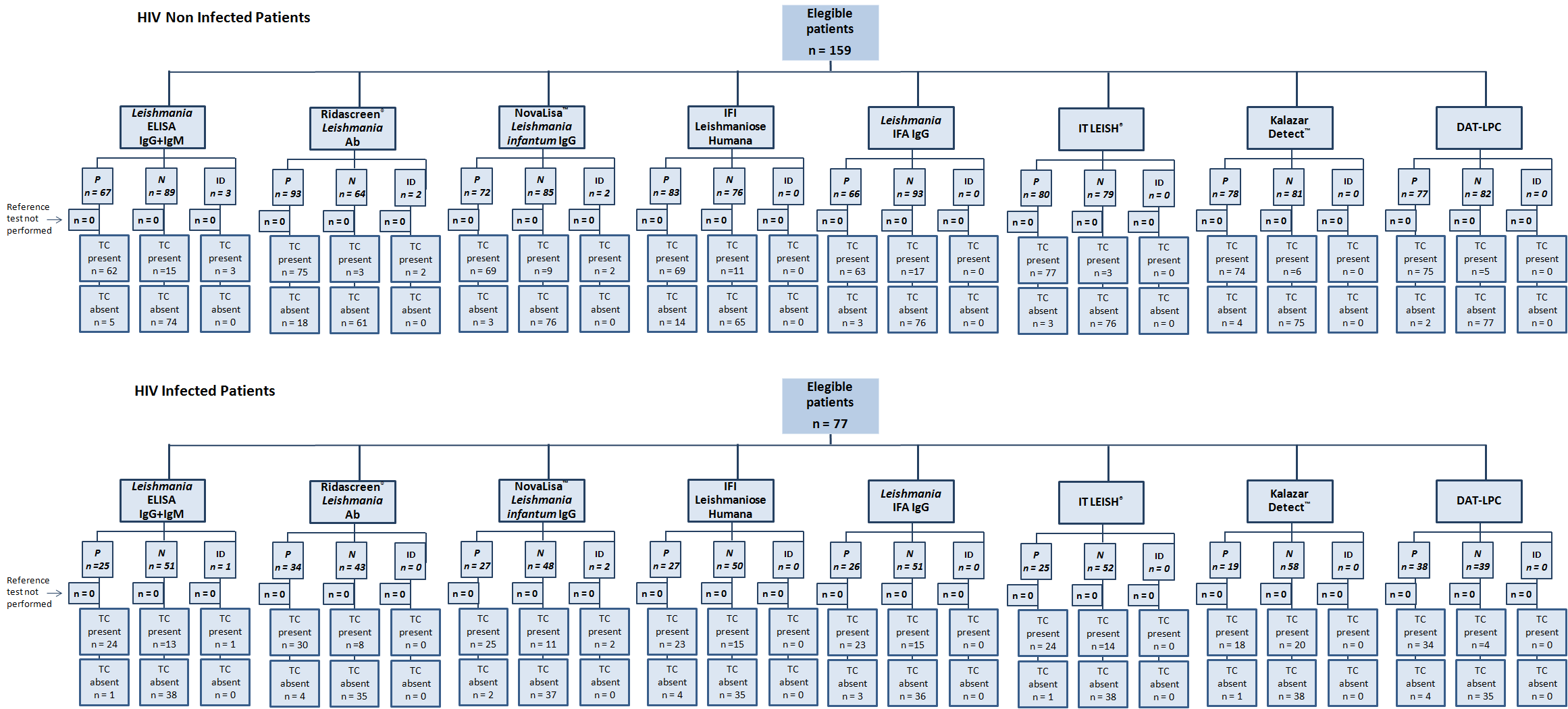

Supplement: S1 Fig — P: positive; N: negative; ID: indeterminate; TC: target condition. (TIF) [file pntd.0007484.s002.tif]
