# Supplementary material for: Performance of serological tests available in Brazil for the diagnosis of human visceral leishmaniasis
Source: PLoS Negl Trop Dis. 2019 Jul 18;13(7):e0007484. doi: 10.1371/journal.pntd.0007484 (PMC6638734; doi:10.1371/journal.pntd.0007484)
Supplement: S1 Table — The concordance analysis was calculated through Cohen kappa index to demonstrate the agreement between the test’s results. (DOCX) [file pntd.0007484.s003.docx]

**S1 Table. Concordance analysis of commercially available diagnostic kits for human visceral leishmaniasis in Brazil.** The concordance analysis was calculated through Cohen kappa index to demonstrate the agreement between the test’s results.

| **Diagnostic Kits** | | **HIV NEGATIVE** | **HIV POSITIVE** |
| --- | --- | --- | --- |
|  |  | **Índice *Kappa***  **IC 95%** | **Índice *Kappa***  **IC 95%** |
| ***Leishmania* ELISA IgG+IgM** | **Ridascreen**^®^ ***Leishmania* Ab** | 0.65 (0.53 - 0.76)^b^ | 0.82 (0.69 - 0.96)^a^ |
|  | **NovaLisa^™^ *Leishmania infantum* IgG** | 0.87 (0.79 - 0.95)^a^ | 0.78 (0.64 - 0.92)^b^ |
|  | **IFI Leishmaniose Humana** | 0.55 (0.42 - 0.68)^c^ | 0.71 (0.54 - 0.88)^b^ |
|  | ***Leishmania* IFA IgG** | 0.80 (0.71 - 0.90)^b^ | 0.79 (0.65 - 0.94)^b^ |
|  | **IT LEISH^®^** | 0.74 (0.64 - 0.85)^b^ | 0.70 (0.53 - 0.87)^b^ |
|  | **Kalazar Detect^™^** | 0.77 (0.67 - 0.87)^b^ | 0.62 (0.43 - 0.81)^b^ |
|  | **DAT - LPC** | 0.76 (0.65 - 0.86)^b^ | 0.68 (0.53 - 0.84)^b^ |
| **Ridascreen**^®^ ***Leishmania* Ab** | **NovaLisa^™^ *Leishmania infantum* IgG** | 0.74 (0.63 - 0.84)^b^ | 0.83 (0.71 - 0.86)^a^ |
|  | **IFI Leishmaniose Humana** | 0.64 (0.52 - 0.76)^b^ | 0.54 (0.36 - 0.73)^c^ |
|  | ***Leishmania* IFA IgG** | 0.63 (0.52 - 0.74)^b^ | 0.73 (0.58 - 0.88)^b^ |
|  | **IT LEISH^®^** | 0.73 (0.63 - 0.84)^b^ | 0.70 (0.54 - 0.86)^b^ |
|  | **Kalazar Detect^™^** | 0.68 (0.57 - 0.80)^b^ | 0.53 (0.35 - 0.71)^c^ |
|  | **DAT - LPC** | 0.72 (0.62 - 0.83)^b^ | 0.79 (0.66 - 0.93)^b^ |
| **NovaLisa^™^ *Leishmania infantum* IgG** | **IFI Leishmaniose Humana** | 0.61 (0.48 - 0.73)^b^ | 0.71 (0.54 - 0.88)^b^ |
|  | ***Leishmania* IFA IgG** | 0.86 (0.78 - 0.94)^a^ | 0.80 (0.65 - 0.94)^b^ |
|  | **IT LEISH^®^** | 0.82 (0.73 - 0.91)^a^ | 0.77 (0.61 - 0.92)^b^ |
|  | **Kalazar Detect^™^** | 0.77 (0.67 - 0.87)^b^ | 0.69 (0.52 - 0.86)^b^ |
|  | **DAT - LPC** | 0.86 (0.78 - 0.94)^a^ | 0.73 (0.57 - 0.88)^b^ |
| **IFI Leishmaniose Humana** | ***Leishmania* IFA IgG** | 0.54 (0.41 - 0.67)^c^ | 0.63 (0.44 - 0.81)^b^ |
|  | **IT LEISH^®^** | 0.69 (0.57 - 0.80)^b^ | 0.59 (0.40 - 0.78)^c^ |
|  | **Kalazar Detect^™^** | 0.64 (0.52 - 0.76)^b^ | 0.57 (0.98 - 0.77)^c^ |
|  | **DAT - LPC** | 0.62 (0.50 - 0.74)^b^ | 0.56 (0.38 - 0.74)^c^ |
| ***Leishmania* IFA IgG** | **IT LEISH^®^** | 0.75 (0.65 - 0.85)^b^ | 0.68 (0.50 - 0.85)^b^ |
|  | **Kalazar Detect^™^** | 0.75 (0.65 - 0.85)^b^ | 0.60 (0.40 - 0.79)^c^ |
|  | **DAT - LPC** | 0.76 (0.66 - 0.86)^b^ | 0.64 (0.47 - 0.80)^b^ |
| **IT LEISH^®^** | **Kalazar Detect^™^** | 0.90 (0.83 - 0.97)^a^ | 0.68 (0.51 - 0.86)^b^ |
|  | **DAT - LPC** | 0.86 (0.78 - 0.94)^a^ | 0.66 (0.50 - 0.82)^b^ |
| **Kalazar Detect^™^** | **DAT - LPC** | 0.81 (0.72 - 0.90)^a^ | 0.45 (0.28 - 0.63)^c^ |

^a^Concordance classified as excellent; ^b^Concordance classified as good; ^c^Concordance classified as moderate
